# Correlative Chemical Imaging to Reveal the Nature of Different Commercial Graphene Materials

**DOI:** 10.1002/smtd.202502344

**Published:** 2026-02-02

**Authors:** Robert Schusterbauer, Paul Mrkwitschka, Mario Sahre, Elena Corrao, Amaia Zurutuza, Alexander Doolin, Francesco Pellegrino, Jörg Radnik, Ievgen S. Donskyi, Vasile‐Dan Hodoroaba

**Affiliations:** ^1^ Federal Institute for Material Research and Testing (BAM), Division 6.1 Surface and Thin Film Analysis Berlin Germany; ^2^ Department of Biology, Chemistry, and Pharmacy Freie Universität Berlin Berlin Germany; ^3^ Department of Chemistry University of Turin Torino Italy; ^4^ Graphenea S.A. San Sebastián Spain; ^5^ Haydale Graphene Industries PLC Ammanford UK

**Keywords:** analytical methods, commercial products, correlative analysis, graphene, surface imaging

## Abstract

Proper physicochemical characterization of advanced materials and complex industrial composites remains a significant challenge, particularly for nanomaterials, whose nanoscale dimensions and mostly complex chemistry challenge the analysis. In this work, we employed a correlative analytical approach that integrates atomic force microscopy (AFM), scanning electron microscopy (SEM) coupled with energy‐dispersive X‐ray spectroscopy (EDS), time‐of‐flight secondary ion mass spectrometry (ToF‐SIMS), Auger electron spectroscopy (AES), and Raman spectroscopy. This combination enables detailed chemical and structural characterization with sub‐micrometer spatial resolution. Three commercial graphene‐based materials of varying complexity were selected and investigated to test the analytical performance of this approach. Furthermore, one of the commercial graphene oxide samples was chemically functionalized via amination and fluorination. This allowed us to assess how surface modifications influence both the material properties and the limits of the applied analytical techniques.

## Graphical abstract

Commercial graphene‐based materials from different sources and of varying complexities were investigated correlatively using commonly employed imaging techniques, including SEM, AFM, ToF‐SIMS, AES, and EDS. ToF‐SIMS combined with SEM was identified as the most effective combination for simultaneously resolving the complex morphology and chemical composition of graphene materials and their mixtures.

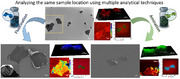

## 1 Introduction

Graphene celebrated its 20th anniversary last year, following the landmark 2004 report by Novoselov and Geim [1]. In the two decades since, its remarkable electronic, [2] mechanical [3] and thermal properties [4] fueled intense research and raised expectations for transformative applications [5]. As with most novel materials, however, the path from discovery to scalable technologies has been gradual [6]. Advances in synthesis [7, 8, 9, 10, 11, 12, 13, 14] and functionalization [15, 16, 17, 18, 19, 20] have yielded a diverse graphene “family”, including derivatives such as graphene nanoribbons, [21] quantum dots [22] and graphene oxide (GO) [23]. This diversity underscores the critical need for rigorous physicochemical characterization [24].

Graphene is now transitioning from a scientific curiosity to an actual industrially relevant material, appearing in composites, [25, 26, 27] coatings [28, 29] and electronic devices [30, 31, 32, 33]. Yet, since it is often integrated in rather small amount with other materials, advanced analysis and quality assurance remain difficult [34, 35, 36]. Surface‐sensitive characterization techniques are central to address these challenges. Imaging methods such as atomic force microscopy (AFM), [37] scanning electron microscopy (SEM) [38] and transmission electron microscopy (TEM) [39, 40] provide morphological insights, while coupling to energy‐dispersive X‐ray spectroscopy (EDS) [41] (in case of SEM or TEM) will add chemical information to the morphological information. However, other chemical imaging methods like Auger electron spectroscopy (AES) and time‐of‐flight secondary ion mass spectrometry (ToF‐SIMS) [42, 43] are often better suited for chemical imaging of 2D materials as they both offer higher lateral resolution, shallower sampling depth and a lower limit of detection [44]. Especially, the superior lateral resolution of these both techniques is their great advantage compared to X‐ray photoelectron spectroscopy (XPS) which is usually central for the chemical analysis of functionalized graphene materials [45]. In addition, Raman spectroscopy is widely used as a complementary method to investigate their chemical and structural properties [46, 47, 48].

It should be noticed that each technique has analytical advantages, but also inherent limitations when used alone, e.g. limited spatial resolution in EDS, high detection limits in AES, or localization challenges in ToF‐SIMS. These critical analytical figures of merit are particularly pronounced for samples with low analyte (light elements/compounds) concentration (<1 wt.%), in soft matrices (such as carbon matrix), and with micro‐scale heterogeneity. In such cases, a combination of several techniques is often needed to draw the full picture [49, 50]. One representative example of such analytically challenging materials is commercial graphene‐containing composites, which typically incorporate only small amounts of graphene (<1 wt.%) dispersed within a carbon‐based matrix, often together in combination with other carbonaceous components [51]. In this context, it is particularly important for materials heterogenous on the micro‐scale to ensure that all results are obtained from the same sample location.

Having this in mind, we selected and imaged the same area of different relevant industrial graphene and graphene‐containing products with the above‐mentioned techniques. Further, we compared the results obtained with different chemical imaging methods and correlated morphological and chemical information by overlaying SEM and ToF_‐_SIMS images. By this methodology the sensitivity of chemical images was enhanced regarding detection of 2D materials on a sub‐micrometer scale in a complex matrix. We were able to further demonstrate that functionalization of graphene flakes after fluorination occurred at defined surface sites and that related trace metals were likewise localized at those positions. Ultimately, we performed the industrial fluorination and amination reaction on lab scale by ourselves with the simplest type of commercial GO flakes to gain a deeper knowledge into the spatial distribution of functionalization.

## 2 Results and Discussion

**FIGURE 1:**
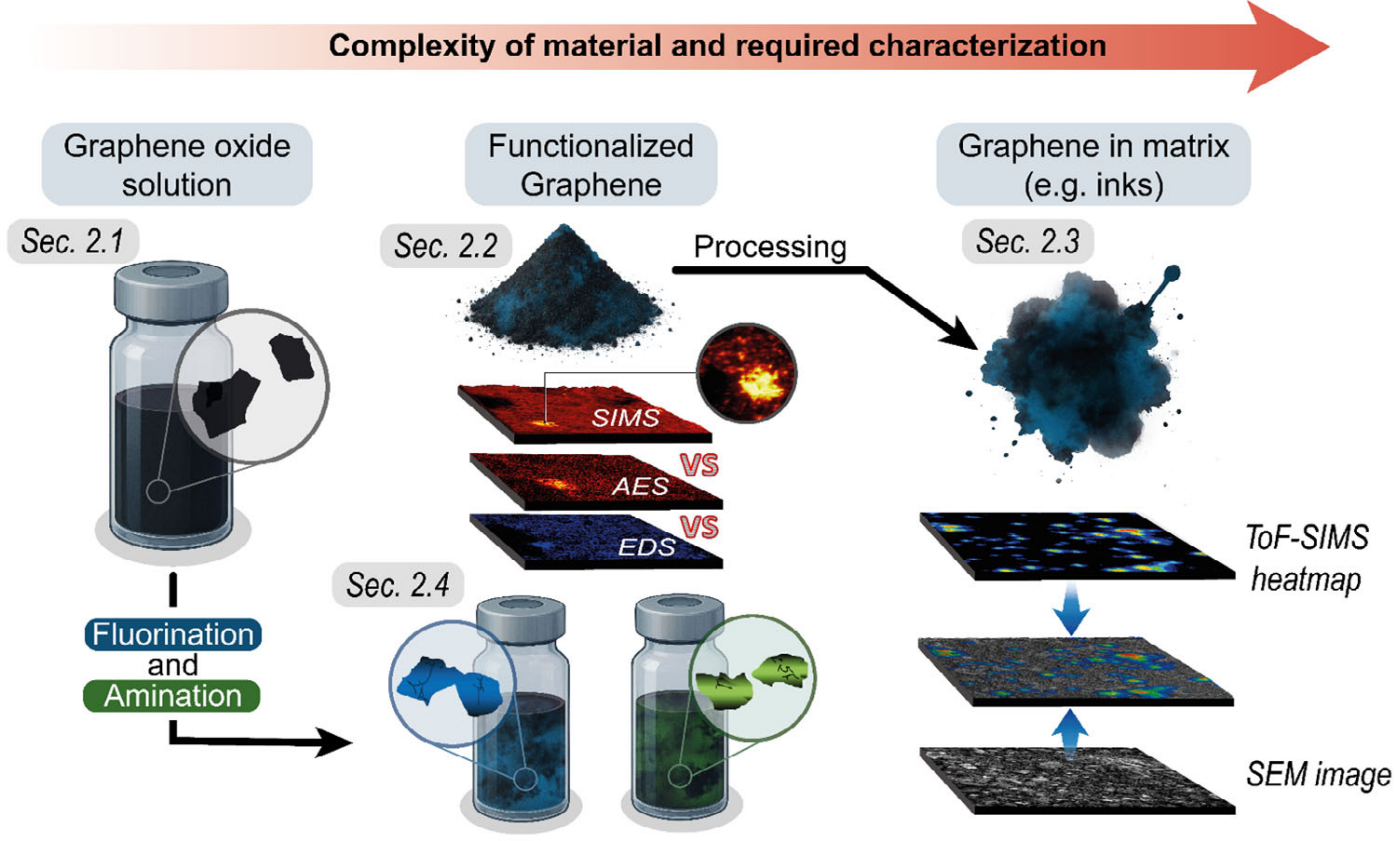
Schematic overview of graphene‐based samples investigated in this study, highlighting their complexity and challenges in characterization.

Within this work, commercial graphene‐containing materials from different sources, different complexities, and tailored for different applications have been investigated (Figure 1). First, unfunctionalized GO (Graphenea, Spain; Section 2.1), functionalized graphene powder (Section 2.2) and the functionalized powder within a commercial ink (Section 2.3, Haydale, fluorinated and aminated graphene powder within ink) have been investigated. In Section 2.2, we compared the three most commonly used chemical imaging techniques (ToF‐SIMS, AES, and EDS). To thoroughly investigate the complex ink matrix, we overlayed ToF‐SIMS and SEM images in Section 2.3. Finally, the unfunctionalized GO flakes were functionalized by us with fluorine and amine groups to get deeper insights into the effect of the functionalization on the analytical challenges and the morphology of the flakes (Section 2.4).

### 2.1 Well‐Defined Monolayer Graphene Oxide (over 98% Monolayers) in µm Range

A commercial solution of GO in water (Graphenea), which is currently used in construction, packaging and filters, was dispensed onto an alignment‐marked native SiO_2_ on Si substrate. Sample preparation is a critical initial step for successful correlative analysis, as it must be tailored to the specific analytical task, and influences the resulting observations. For the purpose of examining the nature of individual graphene oxide flakes, a very low concentration of GO (0.02 g/L) was utilized in a 1:1 water/isopropanol mixture to avoid agglomeration of GO flakes and to minimize coffee ring effects [52]. The marked substrate facilitated the easy finding and precise localization of selected areas necessary for subsequent analyses (Figure S1).

**FIGURE 2:**
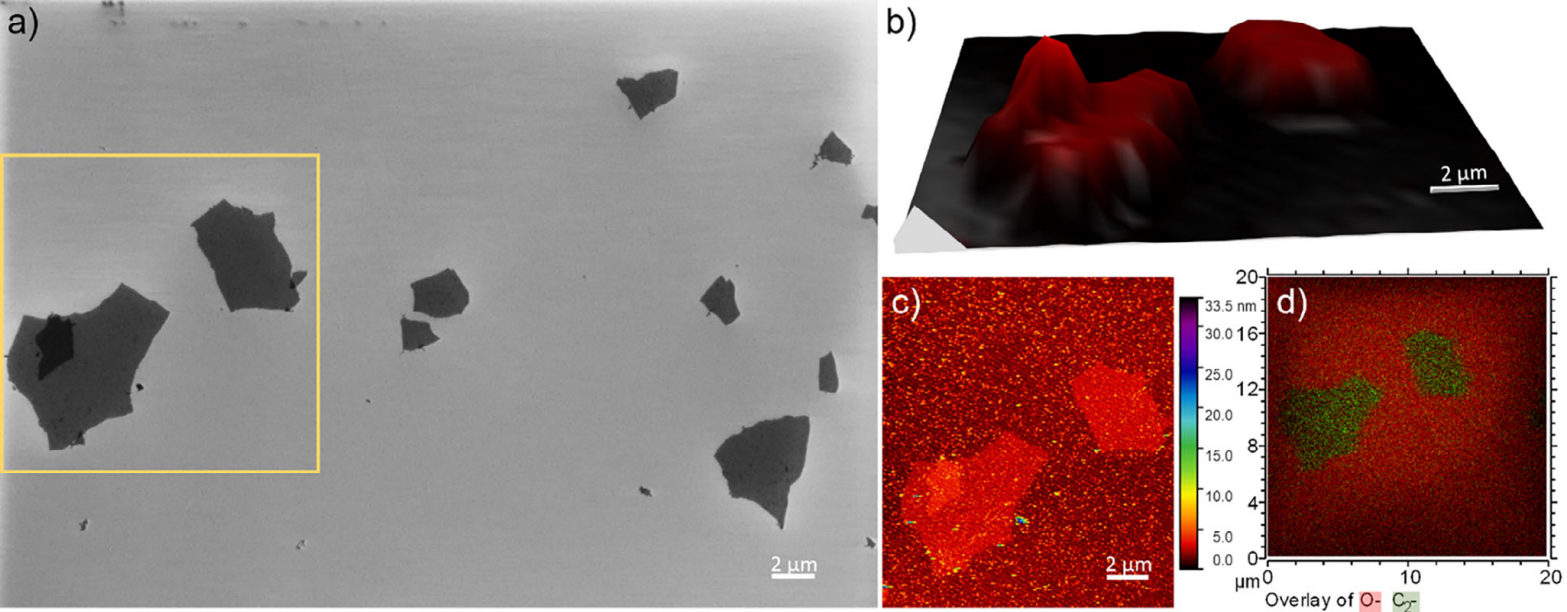
Correlative images of GO flakes deposited on a SiO_2_/Si substrate a) SEM image, b) Raman intensity of G peak scanned over the area in (a) marked with yellow, c) AFM image, and d) ToF‐SIMS overlay image of C_2_− (green) and O− (red) fragments.

Initially, the integrity of the sample after the flake deposition on the substrate was assessed using SEM, which provided an overview image (Figure S1). From this assessment, two flakes with specified areas were selected for further analysis AFM, μ‐Raman spectroscopy, and ToF‐SIMS imaging (Figure 2). AFM provides information about the topography and proves that the detected structures are monolayer graphene sheets which flatly lay on the surface. ToF‐SIMS and Raman spectroscopy imaging exhibit strong correlation with the SEM and AFM images and offer valuable insights into the chemical environment of the prepared GO flakes. While only carbon clusters and C_X_H_Y_‐fragments were identified in ToF‐SIMS spectrum, an I_D_/I_G_ ratio of 1.25 in the Raman spectra (Figure S8) indicates that the point defect distance (L_D_) is below 3 nm.[47] This translates into GO bearing every few carbon atoms either a functional group or a defect. Further, an I_G_‐to‐substrate ratio of 1.5 suggests that the flakes are a monolayer and one secondary flake is overlapping with one of them [53].

Given the clarity of the structural properties of this commercial material (i.e. thickness, chemistry, “simple” and well‐defined), it serves as an excellent reference material for further systematic imaging investigations.

### 2.2 Functionalized Graphene Powder

Graphene powder from another supplier (Haydale), modified through the introduction of fluorine, was wire‐printed [54] onto a Si substrate. Due to the nanoliter volume of the deposited droplets, small, round‐shaped spots of approximately 250 µm in diameter were formed on the surface. Each spot contained many individual flakes (Figure S2), which enabled straightforward identification of the measurement areas.

**FIGURE 3:**
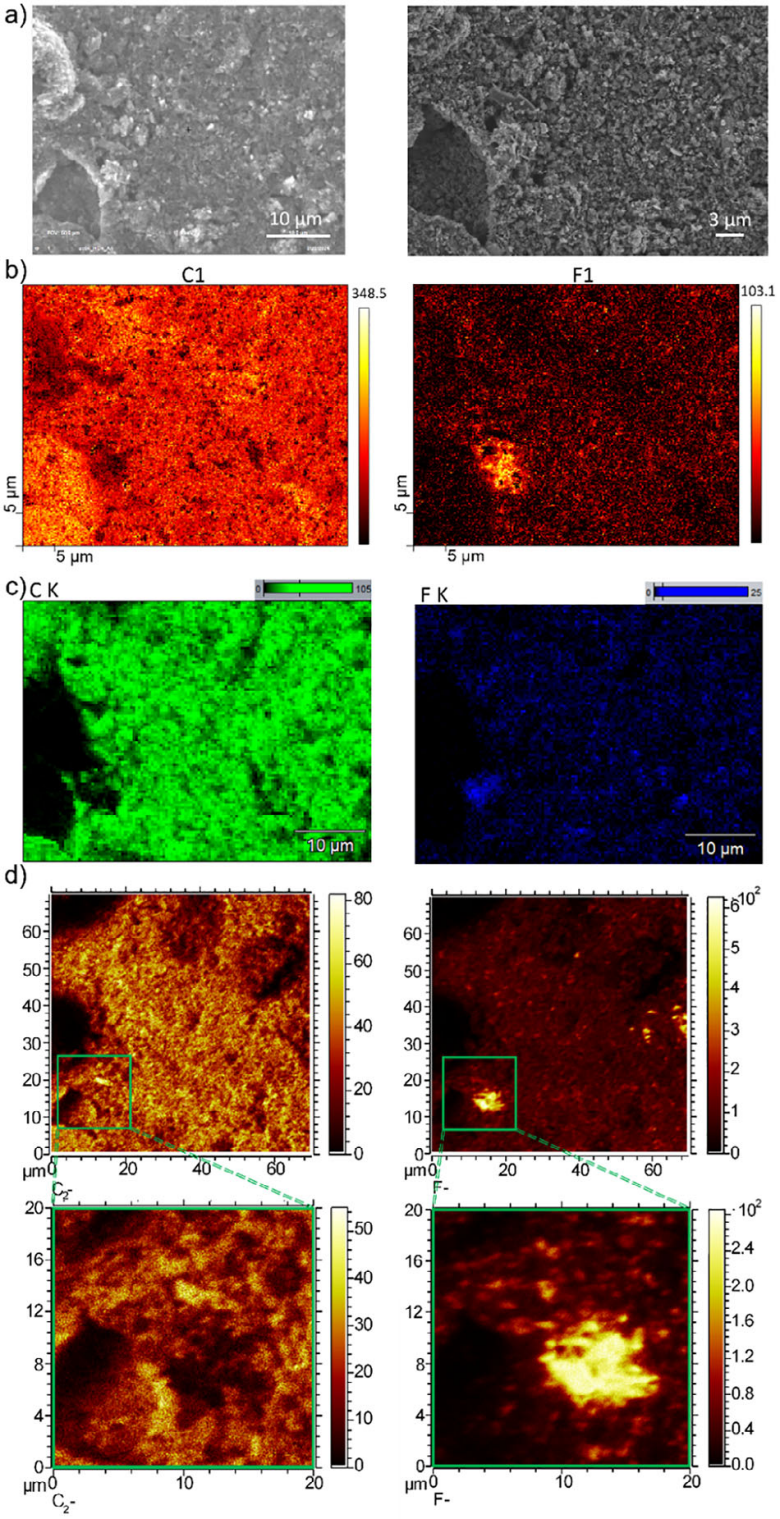
Correlative images of Fluorine‐functionalized graphene powder: a) SEM image, b) AES, c) EDS and d) ToF‐SIMS images of Fluorine and Carbon or C_2_^−^ clusters, respectively.

Imaging the graphene powder using SEM and AES revealed that the material is not homogeneous in particle size nor in the spatial‐surface distribution of functionalization. Notably, one larger particle (of about 10 µm size) displayed a significantly higher density of fluorine‐functionalization (Figure 3b, bottom left). ToF‐SIMS and energy‐dispersive X‐ray spectroscopy (EDS) mappings display similar results of fluorine distribution and further confirm the findings with AES. When comparing the results obtained by these three imaging techniques, SEM/EDS, AES and ToF‐SIMS, it should be noted that AES and ToF‐SIMS offer significantly superior spatial resolutions and ToF‐SIMS the best elemental sensitivity. The more intense F signals observed in the maps obtained with the more surface sensitive techniques AES and ToF‐SIMS compared to EDS suggest an enrichment of F at the surface of the powders [55]. Interestingly, while the carbon map from AES presents adjacent areas brighter, EDS and ToF‐SIMS maps indicate a less pronounced signal in that region. This effect can be explained by the higher sensitivity of AES to the (strong) topography of the flakes, especially caused by shadowing effect, compared with both other techniques.

### 2.3 Inks Containing Functionalized Graphene Powder

Since functionalized powders are typically dispersed within complex matrices in real applications, we further analyzed a commercial conductive ink containing the same powder to test our method in a more realistic scenario.

A commercial resin was selected where the manufacturer mixed a thermoplastic polymer within a carrier solvent (resin) and blended commercially sourced carbon black and plasma functionalized graphene powder (Section 2.2) together to formulate a functional ink. The loading ratio of resin to carbon black to functionalized graphene for each ink was identical, with deviation only to the surface functionality of the graphene material itself. Conductive graphene inks have potential applications within the aviation industry, as either coatings or composites, to dissipate the energy from lightning strikes. The quality and reproducibility of the large‐scale coating is of high importance.

Analyzing this ink poses a significant challenge as the material matrix consists of three different carbon‐based materials, with the final composite material being a black, sticky product with a highly complex morphology and chemistry.

**FIGURE 4:**
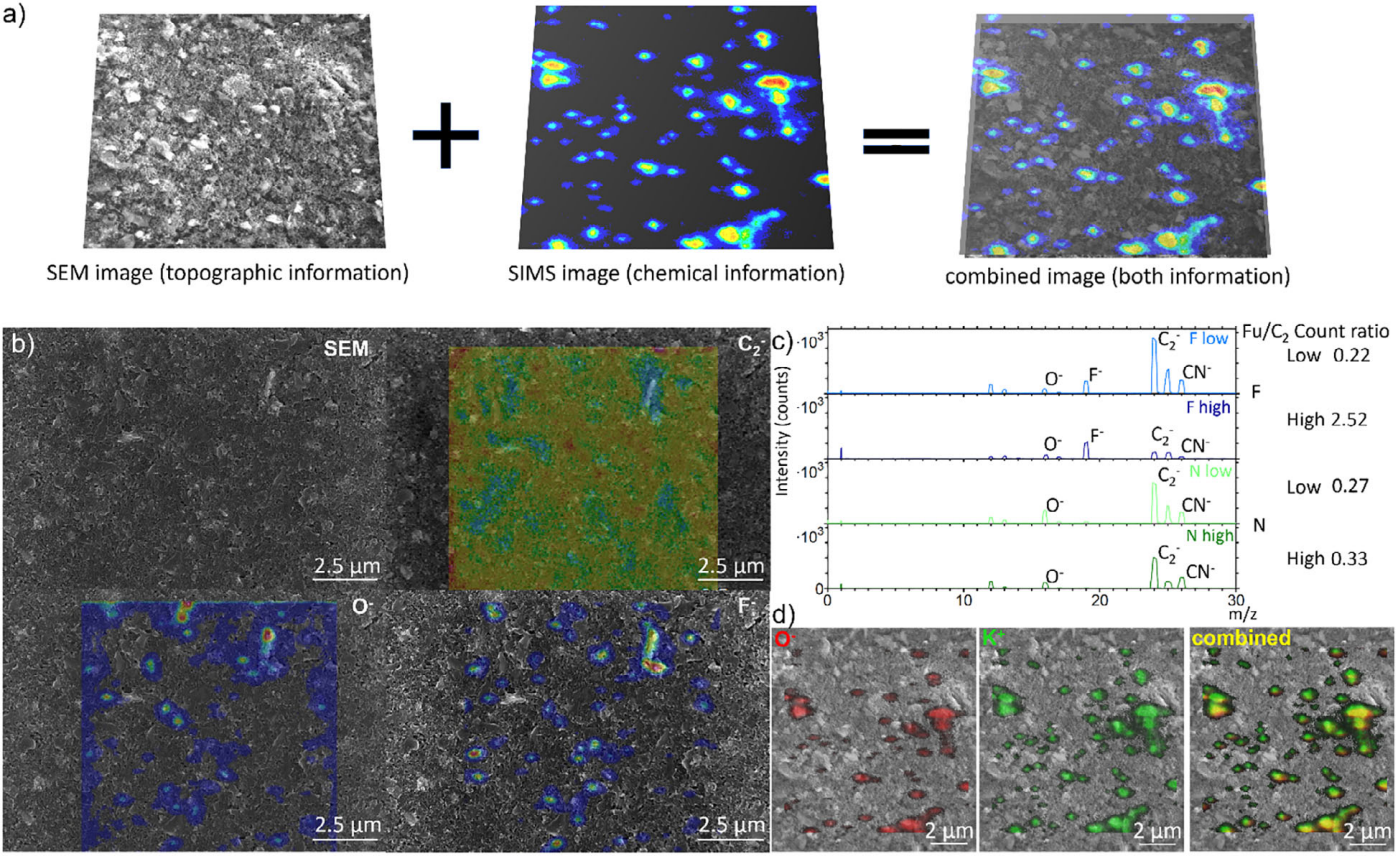
Functionalized graphene in an ink matrix a) schematic representation of the combination of SEM and ToF‐SIMS image b) SEM image combined with ToF‐SIMS heatmaps of low F‐functionalized ink (colors are displayed from low intensity “blue” to high intensity “red”; no signal is displayed as transparent), c) ToF‐SIMS negative mode spectra of N‐ and F‐functionalized inks with low and high degree of functionalization, the ratio of functionalization fragment (Fu) to C_2_ and d) SEM image overlayed with ToF‐SIMS in negative (O^−^) and positive (K^+^) mode and the combination of both species from high N‐functionalized ink.

SEM micrographs suggested that the functionalized graphene flakes, clearly visible across the scanned ink areas, are uniformly distributed within the carbon black ink matrix. Confirmation could be found by correlatively imaging one area with ToF‐SIMS to the SEM image (Figure 4a). Figure 4b shows a heatmap that overlays various fragments detected by ToF‐SIMS with the SEM image. It, clearly illustrates that fluorine, solely associated with the graphene flakes, is homogeneously embedded within the matrix volume.

EDS mapping of fluorine taken at 10 kV could be correlated with the findings above, though with somewhat poorer resolution compared to ToF‐SIMS (Figure S3a,b). Notably, AES analysis of the same area did not reveal fluorine (Figure S4). This can be attributed, in part, to the much higher detection limit of AES (of roughly 1 at‐%), or possible due to the strong surface morphology and/or beam damage due to the point analysis (only possible on the small visible structures), see Figure S4, compared to ToF‐SIMS (0.01 at‐%) but also to the somewhat deeper presence of the same flakes beyond the top surface of the sample. The latter assumption is supported by the fact that EDS with its micro‐sub‐micro spatial resolution and relatively high limits of detection (roughly 1 at‐%) would be able to sense the fluorine functionalisation on those flakes rather embedded in the sample matrix (with an arbitrary spatial orientation) which only partly “stick out” at the top surface of the sample.

To accurately correlate the information obtained from the ToF‐SIMS maps with the SEM micrographs for such small measurement areas on the micrometer scale, the ToF‐SIMS analysis was performed first. This ensured precise alignment of both datasets. The exact location of the ToF‐SIMS ‐scanned area was then identified in the SEM by tracking the bismuth (Bi) signal in the EDS, originating from Bi ions implanted by the liquid metal ion bismuth gun (Figure S3c,d).

In addition to fluorine fragments detected in the ToF‐SIMS spectra, oxygen fragments were also found to derive solely from the graphene flakes. Most of them shared similar spatial positioning. However, the intensity distribution of these fragments varied. The instances where only oxygen was detected suggested that functionalization reactions preferentially occur at specific sites. It was observed that some flakes possessed higher degrees of functionalization than others, indicating variability in the functionalization process (as also analysed in the starting material). The heatmaps of carbon clusters and C_X_H_Y_‐fragments show signals across the whole measured area even though areas of graphene flakes seem to be less pronounced than the rest.

In addition to the ink with fluorinated graphene flakes of low functionalization degree (Figure 4b), the producer also prepared an ink with a higher fluorine content. Furthermore, two inks containing amine‐functionalized graphene flakes were provided. One of these had a low degree of functionalization, and the other a high degree. These three additional samples were also analyzed following the same imaging correlative approach as described above (Figure 4c).

**FIGURE 5:**
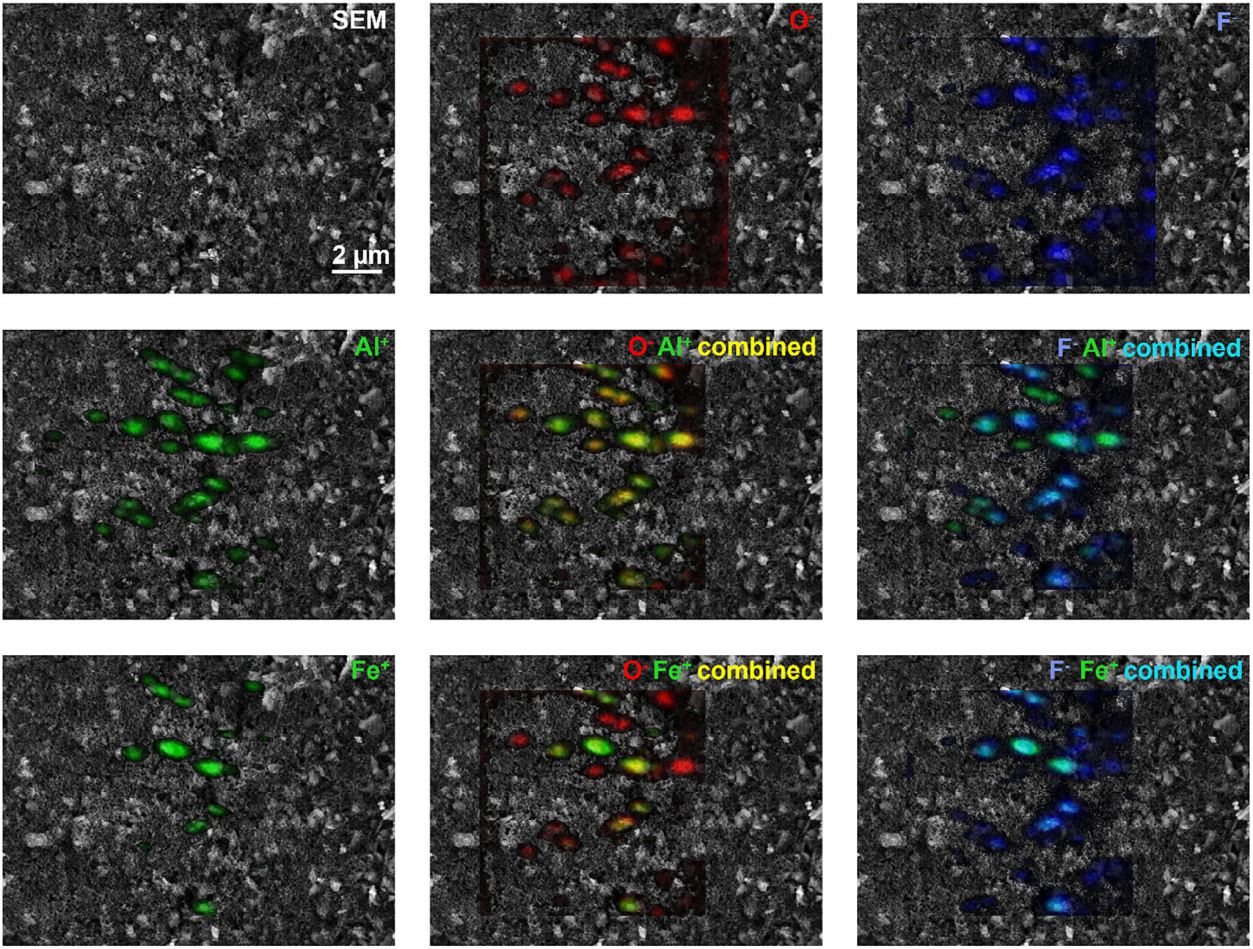
ToF‐SIMS images of highly fluorinated ink showing different fragments (trace metal impurities and F^−^ and O^−^) measured in positive and negative mode combined with SEM image. Areas where metal and O^−^ are overlayed are displayed in yellow, while the metal and F^−^ overlay is shown in turquoise.

The distribution of flakes in the ink with higher fluorine content appeared similar to the low‐fluorine version and covered approximately 10% of the surface (as shown by the F^−^ signal in Figure 5). However, the increased fluorine content was reflected in the intensity of the F^−^ signal in the mass spectra measured under the same conditions. The relative intensity compared to the C_2_^−^ signal rose significantly from 0.22 to 2.52.

Fragments from the amine‐functionalized graphene flakes were more difficult to identify, as the characteristic CN^−^ fragment is also present in the carbon black matrix of the ink. Nonetheless, a slight increase in the CN^−^ signal relative to the C_2_^−^ signal from the low to the highly functionalized ink suggests an increased degree of functionalization. The locations of the graphene flakes could still be traced via the O^−^ signal (Figure 4d).

Moreover, traces of elements such as potassium (K), aluminium (Al) and iron (Fe) from the production process were localized at flake sites from ToF‐SIMS measurements in positive mode. Combining positive and negative mode, ToF‐SIMS results revealed that metal impurities are associated with the graphene flakes, as they localized alongside O^−^ fragments. Figure 4d displays the overlay of the O^−^ and K^+^ signals revealing strong spatial correlation, with overlapping regions appearing yellow (red for O^−^ and green for K^+^).

Notably, similar to the functional groups, these impurities were detected on various sides and edges of the flakes. This phenomenon was examined in more detail for the ink containing highly fluorinated graphene flakes (Figure 5). Even at a brief glance, differences in the spatial distribution of K, Al, Fe, and Na were evident (Figure S5). When comparing Al^+^ and Fe^+^ signals with the F^−^ and O^−^ signals, aluminum appears to align more closely with oxygen, while iron is more strongly associated with fluorine (Figure 5). This finding is clearly visible in the overlay images, demonstrating the power of correlative imaging analysis. Aluminum and oxygen signals showed strong co‐localization, resulting in a yellow color from the overlap of green (Al^+^) and red (O^−^). In contrast, the overlap between aluminum and fluorine was less pronounced, producing only a faint turquoise hue (green Al^+^ and blue F^−^), indicating weaker spatial correlation. Iron, in contrast, exhibited pronounced co‐localization with fluorine, producing a more intense turquoise signal. Only minimal overlap with oxygen was observed, reflected by the weak yellow contribution in those areas.

Sodium and potassium exhibited a distribution pattern similar to that of aluminum, with slight variations in signal intensity from flake to flake. This observation suggests that these three metals may be present on the graphene flakes as metal oxides. The correlation between Fe and F is not straightforward. Recently published XPS data did not support the presence of C─F bonding as the sole chemical motif [56]. In contrast, the spatial analysis presented here reveals a clear co‐localization of Fe and F. However, it must be noted that XPS is not able to determine small amounts of inorganic F in presence of a dominant amount of organic F with a percentage higher than 95%.

### 2.4 Functionalization of Well‐Defined Monolayer Graphene Oxide

**FIGURE 6:**
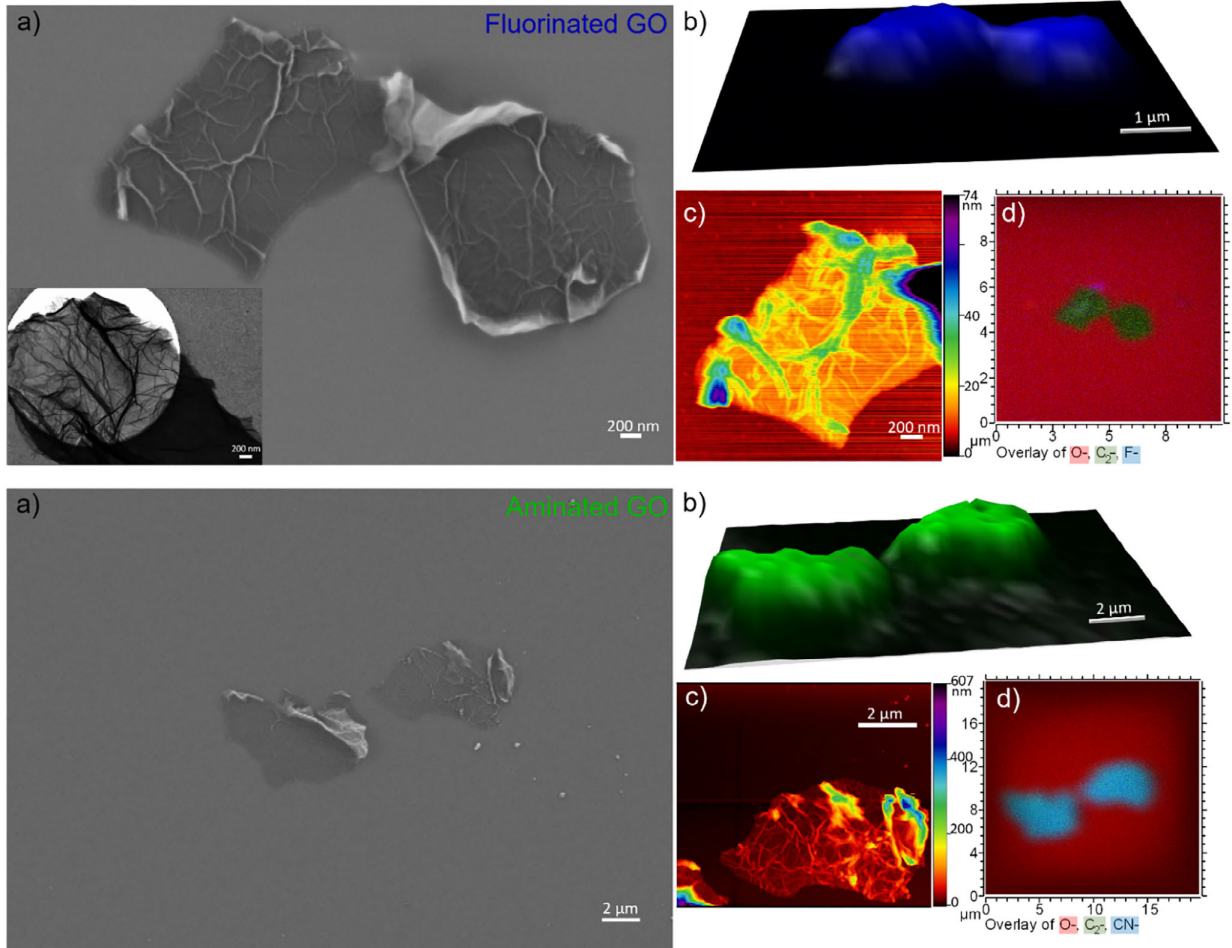
Correlative images of fluorine (top) and amine (bottom) functionalized GO flakes (see same order of imaging methods for top and bottom, a), b), c), d)): a) SEM image (inset in upper image: STEM‐in‐SEM image), b) Raman intensity of G peak scanned over the area in (a), c) AFM image and d) ToF‐SIMS overlay image of C^−^ (green), O^−^ (red), and F^−^ or CN^−^ (respectively, blue) fragments. If green and blue overlay a turquoise color appears.

For gaining deeper understanding of the industrial functionalized material, well‐defined GO (Section 2.1) was functionalized in our lab with fluorine and amines using wet chemistry. The resulting materials were investigated following the same analysis workflow as the unfunctionalized precursor GO. SEM and AFM images (Figure 6a,b) exhibited a drastic change in morphology. The single GO layers stacked and folded building vein‐like structures on the sheets upon reaction. Same was observed with SEM being operated in the transmission mode (inset Figure 6a). At the flat regions of the sheets, the height is ∼5 nm in height. The vein‐like structures are building up to 100 nm in height, according to the AFM imaging in Figure 6c, Figure S6. Both fluorination and amination reactions produce vein‐like structures and enlarged merged sheets (Figure S7) and indicate that the chemical functionalization step strongly affects the stacking behavior of GO. Further work will be required to determine how reaction parameters influence this process. The changes in surface chemistry are studied by Raman spectroscopy/imaging and ToF‐SIMS. Functionalization causes the sheets to thicken and lose their flat morphology. Consequently, the G band Raman intensity no longer reflects the true layer thickness, and the 3D Raman mapping images do not retain height information. However, slight shifts in the Raman spectra, that also reported in the literature, [57, 58] indicate the successful functionalization of the flakes. The G band, located at 1598 cm^−1^ for unfunctionalized GO, shifts to 1607 cm^−1^ upon fluorination (Figure S8). This blue shift reflects phonon stiffening due to a higher strain in the sp^2^ lattice from C─F bonding. Meanwhile, the G band of the aminated product has a red shift to 1593 cm^−1^ after reaction as the reagents partially reduce the hydroxy and epoxide groups. Additionally, whereas fluorine substituents withdraw electron density from the GO sp^2^ lattice, amine groups donate electron density into it.

ToF‐SIMS further confirmed the successful functionalization. Upon functionalization, F^−^ and CN^−^ fragments can be found on the surface of the fluorinated and aminated flakes, respectively. Both fragments were not seen on the unfunctionalized flakes (Figure S9). Interestingly, the fluorine signal shows enhanced intensity on the left graphene flake at the large “vein” and on the edge of the sheet, while the CN^−^ signal for the aminated sample is present across the whole flake. These results are consistent with the observations made for the industrially functionalized graphene (Section 2.2; Section 2.3), where the fluorine content varies from flake to flake and is also heterogeneously distributed across individual flakes.

**FIGURE 7:**
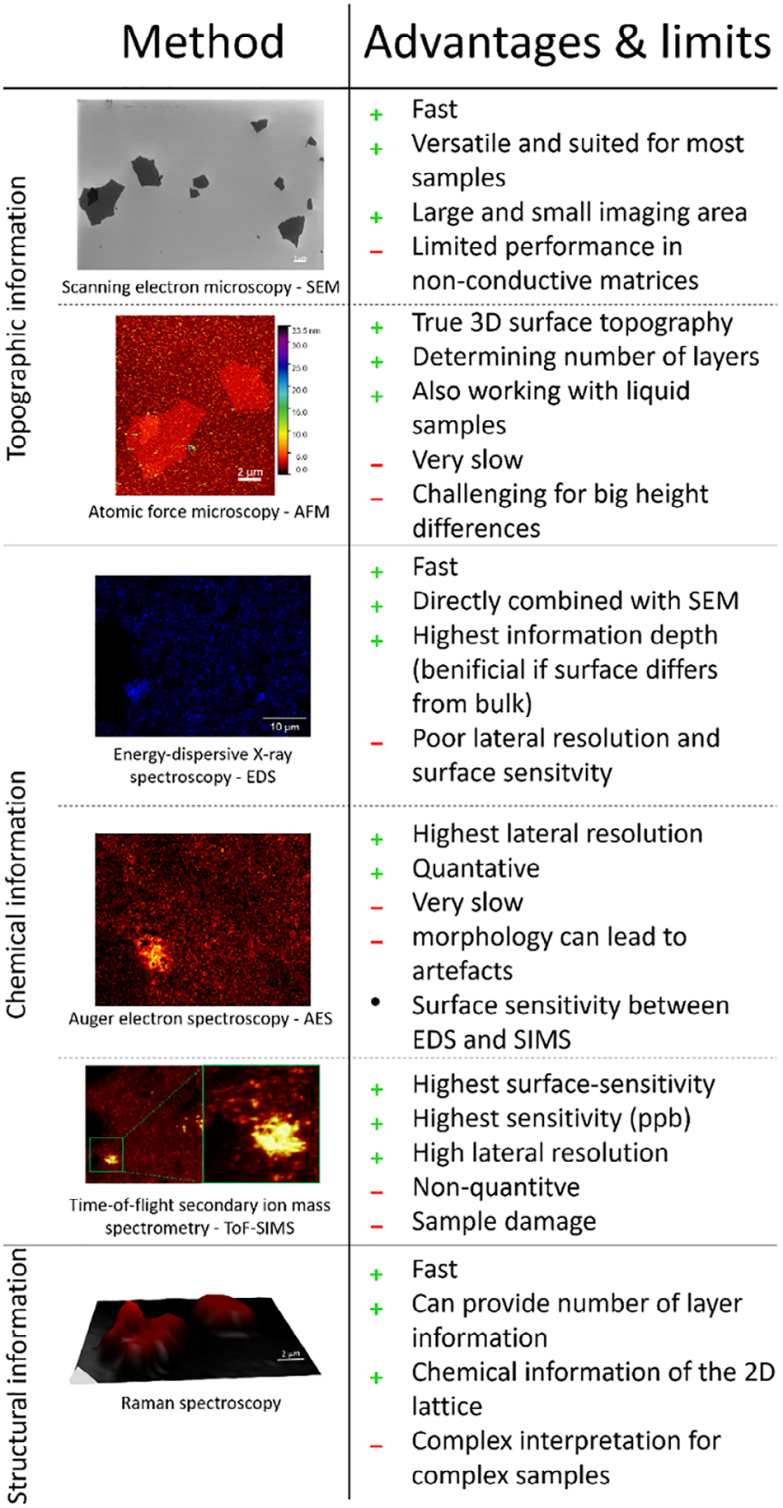
Comparison of the different imaging techniques and their advantages and disadvantages at the analysis of 2D materials.

As discussed, each analytical technique exhibits distinct advantages and limitations (Figure 7). A comparison between the three chemical analysis techniques, ToF‐SIMS, EDS and AES, demonstrates that different parameters play a crucial role when characterizing 2D materials or 2D materials embedded in complex matrices. The surface sensitivity (ToF‐SIMS > AES > EDS), lateral resolution (AES > ToF‐SIMS > EDS) as well as the limit of detection (ToF‐SIMS < AES ≈ EDS) are the most relevant analytical figures of merit for such measurements. Among the techniques evaluated, ToF‐SIMS showed the highest capability for detecting fluorine and amine functionalization on graphene flakes. In combination with SEM imaging, this approach was suitable to track functionalized graphene flakes as well as metal impurities in complex matrices. On the other side, SEM/EDS is the fastest analytical imaging tool, with rough but clear information on the spatial distribution of the main elements and impurities. Thus, the analytical value of correlating and complementing the information from different methods becomes clear, an analytical approach which we strongly recommend.

## 3 Conclusion

In this study, we systematically combined several surface sensitive imaging techniques (AFM, SEM with EDS, AES, ToF‐SIMS, and Raman spectroscopy) to achieve a correlative characterization of industrial graphene‐based materials with high spatial resolution down to the nanometer range. Through analysis of unfunctionalized GO, chemically functionalized graphene powders, and highly complex graphene composite inks, we demonstrate how complementary methods overcome the individual limitations of each technique. The overlay of chemical and morphological maps proved highly effective in the identification of graphene flakes in complex matrices and in the detection of functional groups and trace metal impurities at specific flake sites. Furthermore, we performed the industrial fluorination and amination reactions on well‐defined monolayer GO and demonstrated their impact on flake morphology, surface chemistry and spatial heterogeneity. These results highlight the importance of correlative analytics in advanced graphene materials for industrial quality control.

## Conflicts of Interest

The authors declare no conflict of interest.

## Supporting information




**Supporting File**: smtd70529‐sup‐0001‐SuppMat.docx.

## Data Availability

The data that support the findings of this study are available in the supplementary material of this article.
