# Supplementary material for: Correlative Chemical Imaging to Reveal the Nature of Different Commercial Graphene Materials
Source: Small Methods. 2026 Feb 2;10(9):e02344. doi: 10.1002/smtd.202502344 (PMC13159395; doi:10.1002/smtd.202502344)
Supplement: Supplementary file 1 — Supporting File: smtd70529‐sup‐0001‐SuppMat.docx. [file SMTD-10-e02344-s001.docx]

**Correlative chemical imaging to reveal the nature of different commercial graphene materials**

Robert Schusterbauer^a,b^, Paul Mrkwitschka^a^, Mario Sahre^a^, Elena Corrao^c^, Amaia Zurutuza^d^, Alexander Doolin^e^, Francesco Pellegrino^c^, Jörg Radnik^a^, Ievgen S. Donskyi^a,b*^, Vasile-Dan Hodoroaba^a*^

[a] R. Schusterbauer, P. Mrkwitschka, M. Sahre, Dr. J. Radnik, Dr. I. S. Donskyi*, Dr. V.-D. Hodoroaba*
Federal Institute for Material Research and Testing (BAM)
Division 6.1 Surface and thin film analysis
Unter den Eichen 44–46, 12203 Berlin (Germany)
E-mail: dan.hodoroaba@bam.de

[b] R. Schusterbauer, Dr. I. S. Donskyi*
Department of Biology, Chemistry, and Pharmacy
Freie Universität Berlin
Altensteinstraße 23a, 14105 Berlin (Germany)

[c] Dr. E. Corrao, Dr. F. Pellegrino
University of Turin
Department of Chemistry
Via Giuria 7, 10125 Torino, Italy

[d] Dr. A. Zurutuza
Graphenea S.A.
Paseo Mikeletegi 83, 20009 San Sebastián, Spain

[e] Dr. A. Doolin
Haydale Graphene Industries PLC
Clos Fferws, Parc Hendre, Ammanford SA18 3BL, United Kingdom

Supplementary Information

Figures


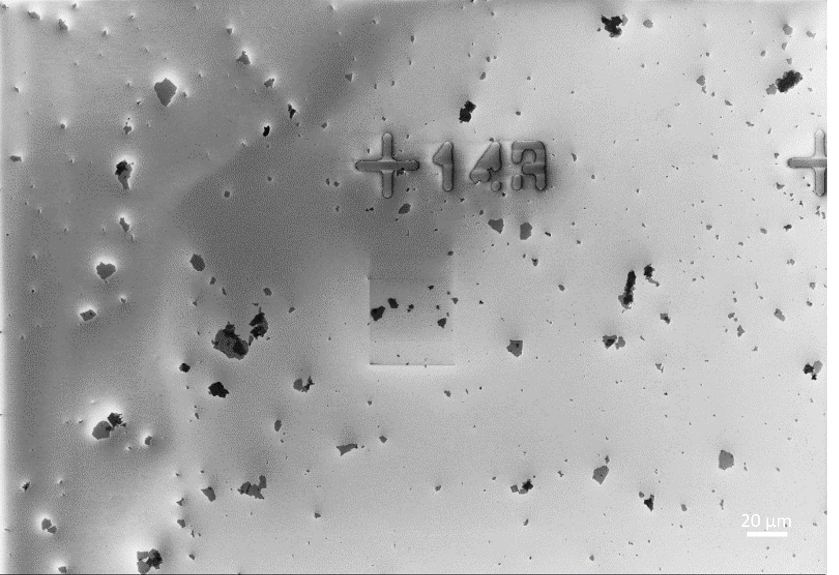


**Figure S1**: Low-magnification, overview SEM micrograph of single layer graphene oxide flakes with marking.


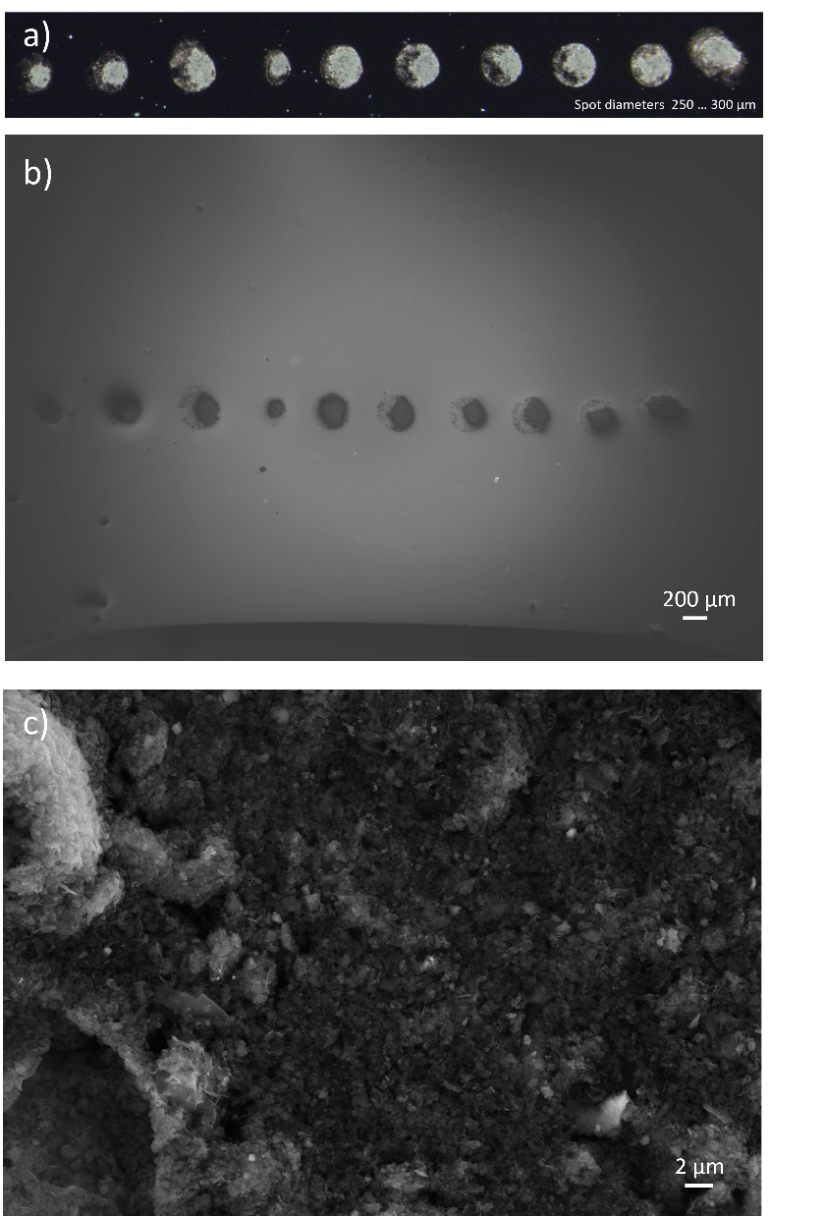


**Figure S2**: a) Light microscopy image of wire-printed^[54]^ spots of fluorinated graphene powder b) SEM image of the same area as in (a) and c) close-up image on spot number 6 which was used for correlative analysis.


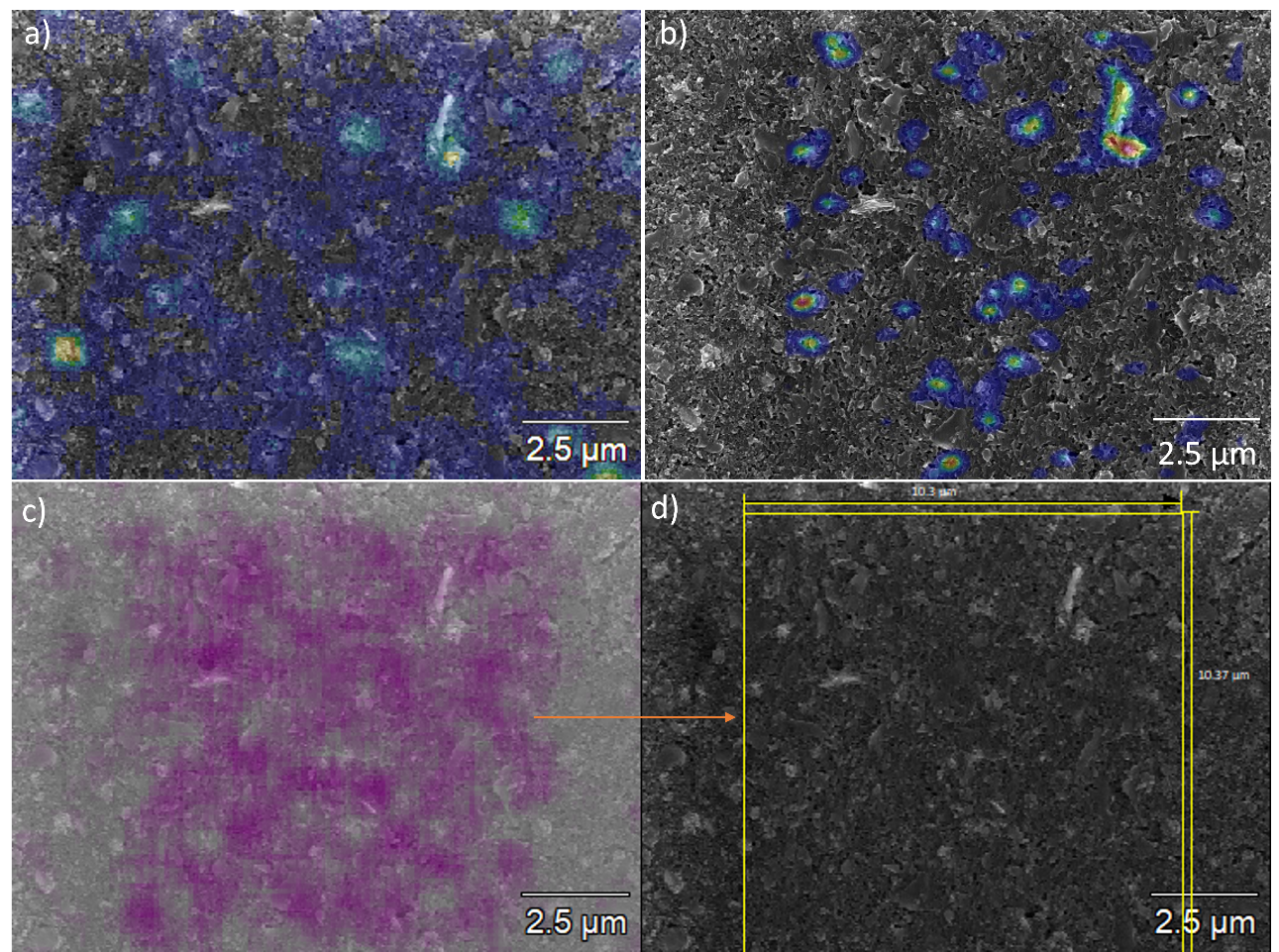


**Figure S3**: Comparison of a) F Kα EDS map and b) ToF-SIMS F^-^ image (both on SEM image). c) Bi Mα EDS map of implanted Bi ions from ToF-SIMS measurement and d) from (c) derived measurement area shown as a yellow box with distances.


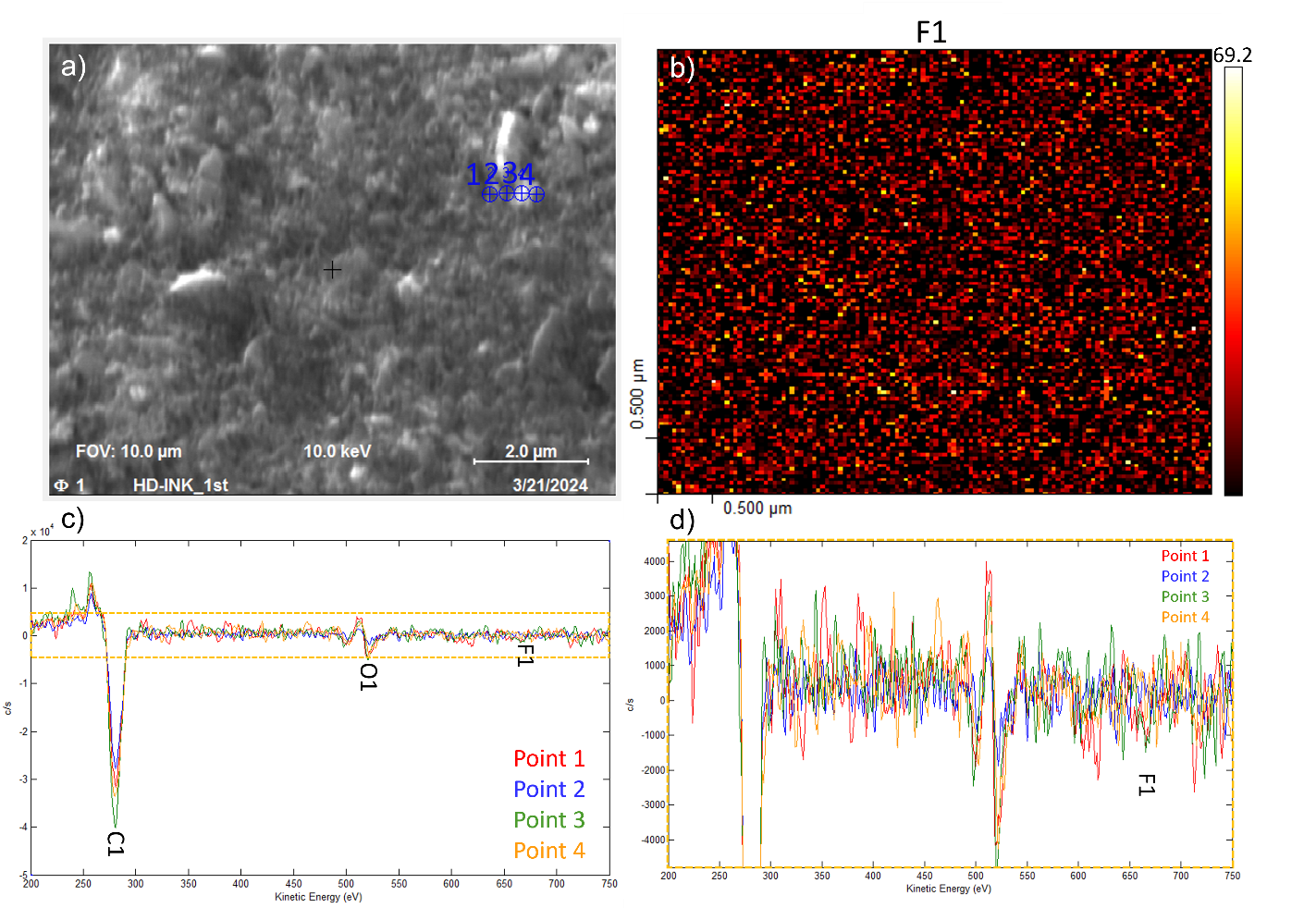


**Figure S4:** a) SEM micrograph with points measured by AES, b) AES map of part of the region shown in (a), c) AES spectrum of the points from (a) and d) zoom-in of marked region in (c).


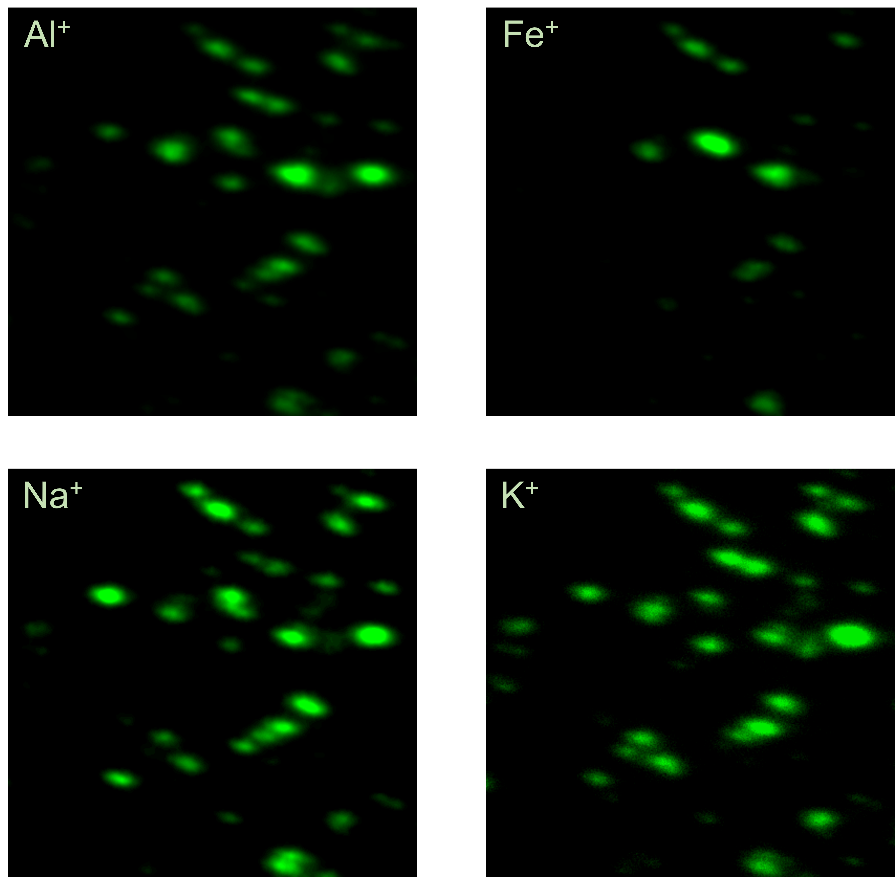


**Figure S5**: Positive ToF-SIMS images of different trace metal ions found in the highly fluorinated ink.


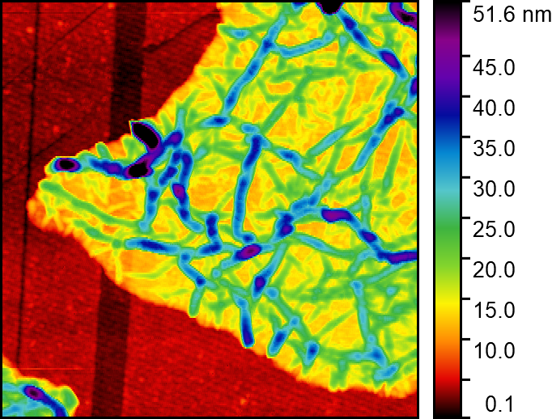


**Figure S6**: High-magnification AFM image of the right aminated flake to observe the “vein”-like structures.


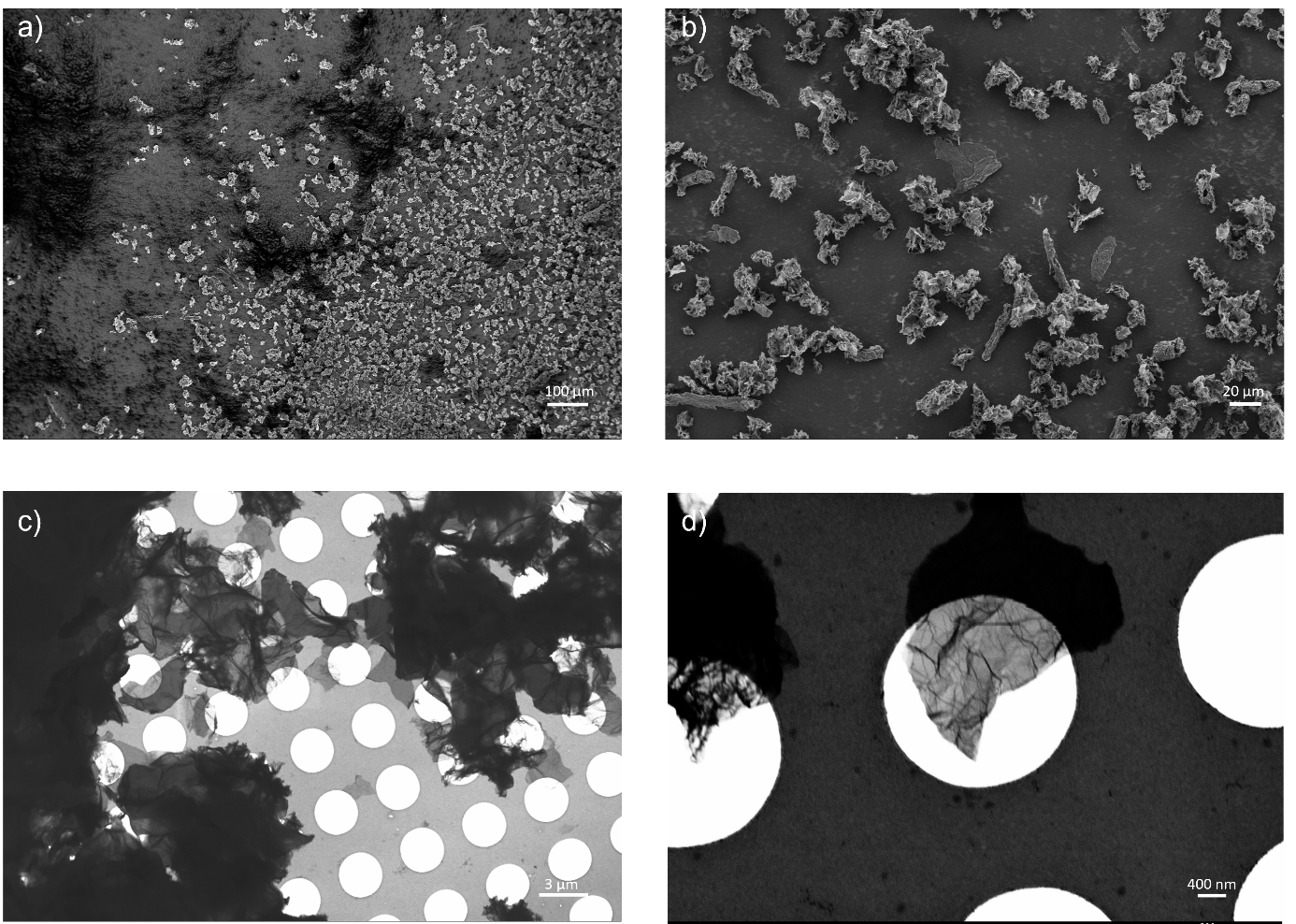


**Figure S7**: SEM images with low (a) and high (b) magnification of functionalized graphene oxide flakes. Imaged as received and deposited dry on carbon tape. STEM-in-SEM with low (c) and high (d) magnification of functionalized flakes drop-casted on TEM grid.


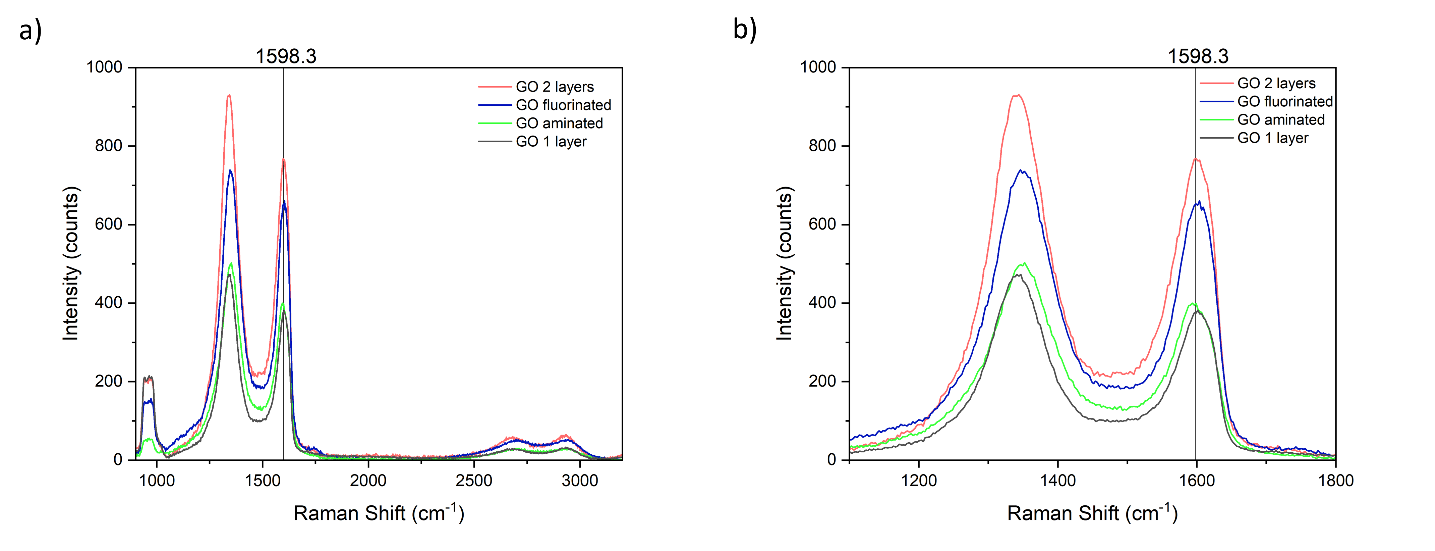


**Figure S8**: a) Raman spectra of monolayer and double layer region of graphene oxide and spectra of fluorinated and aminated graphene oxide samples deposited on a silicon substrate, b) Zoom-in of (a).


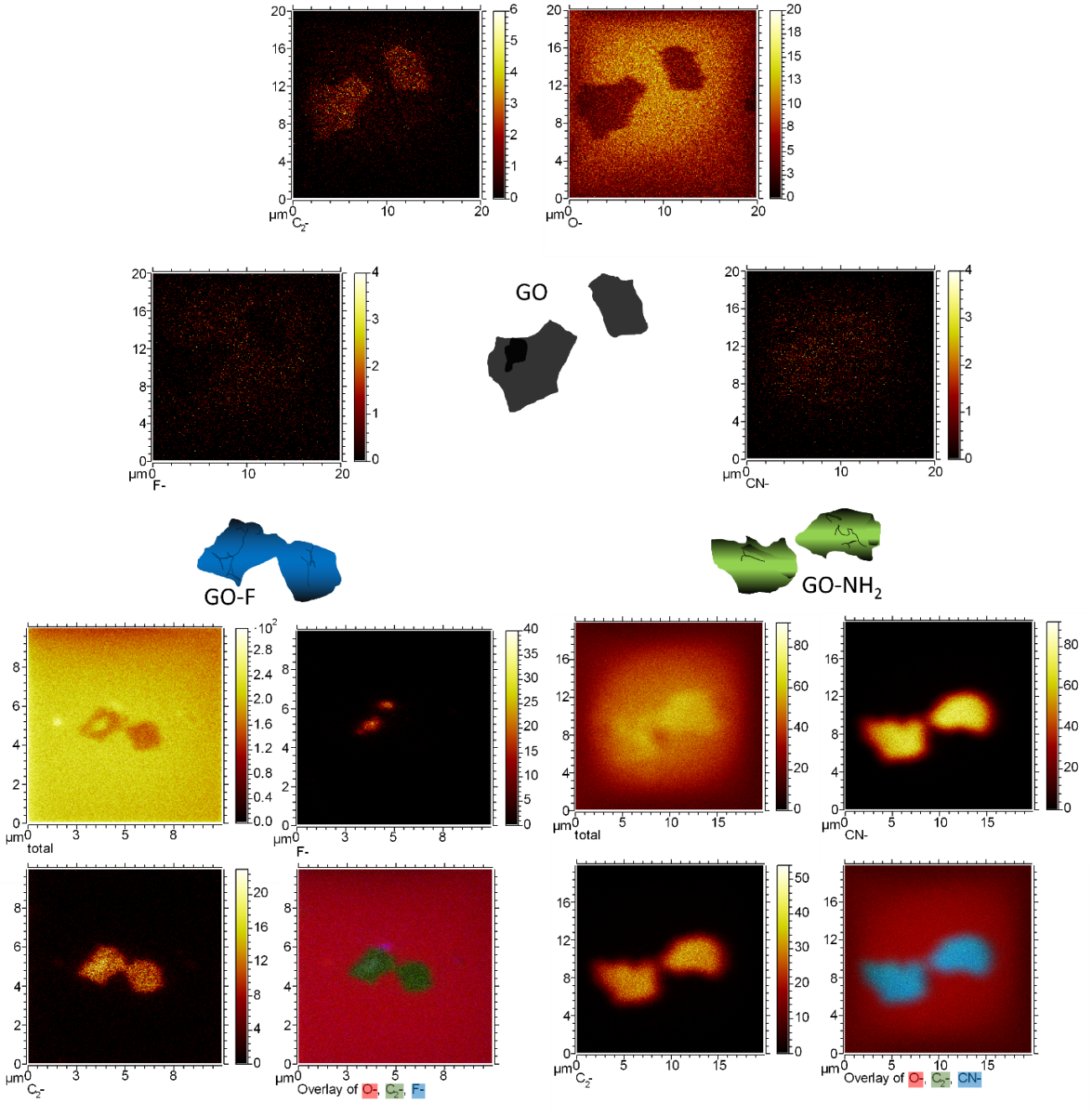


**Figure S9**: ToF-SIMS negative images of graphene oxide flakes and functionalized (fluorinated and aminated) graphene oxide flakes.

Synthetic procedures

For the synthesis of the fluorinated GO flakes (internal name: BAM_GO_F_HC), 40 mL of a 4 mg/mL suspension of Graphene Oxide (Graphenea, was combined with 37 mL of water, ensuring that the final volume reached 81 mL when 4 mL of 48% HF was added. The mixture was placed in a Teflon liner and subjected to hydrothermal synthesis at 180 °C for 30 hours. The product was processed following the same procedure as above: after cooling to room temperature, the autoclave was opened, and the product was washed multiple times with Milli-Q water.

For the synthesis of the aminated GO flakes (internal name: BAM_GO_NH_2__HC), Graphene oxide (Graphenea, 22.5 ml of the 4 mg/ml suspension) was diluted in deionized water (final volume 126 mL) under magnetic stirring. Aqueous ammonia (300 mmol, 28 wt-%) and sodium bisulfite (18 mmol) were subsequently added to the dispersion. The mixture was sealed in a Teflon-lined stainless-steel autoclave and heated at 170 °C for 10 h. After natural cooling to room temperature, the product was collected by centrifugation and washed three times with deionized water to remove residual reagents. The obtained material, denoted BAM_GO_NH_2__HC, was washed several times with Milli-Q water.

Measurement procedures

ToF-SIMS: ToF-SIMS analysis was performed using a TOF-SIMS M VI instrument (IONTOF GmbH, Münster, Germany). The samples were analyzed at room temperature. Measurements were conducted in spectrometry mode with a 25 keV Bi₃⁺ primary ion beam in negative and positive polarity. Different field of views (FoV) measuring between 10 x 10 and 70 × 70 μm^2^ were rastered in random mode with 2048 × 2048 pixels, acquiring one shot per pixel. Spectra were calibrated using typical organic fragments (C^-^ at 12.01, O^-^ at 16.00, C₂^-^ at 24.02, C_3_^-^ at 36.03 m/z for negative and CH^+^_2_ (14.02), C_2_H_4_^+^ (28.03), C_3_H_6_^+^ (42.05), and C_4_H_8_^+^ (56.06) for positive mode). The images were binned with different factors between 4 and 64 pixels.

EDS: An SDD EDS detector (Thermo Fisher Scientific, USA) with a 100 mm^2^ nominal area was used with a Supra 40 scanning electron microscope (Zeiss, Oberkochen, Germany). Spectra were collected at 5, 10 and 15 kV beam voltage.

AFM: Surface topographies were acquired by atomic force microscopy (AFM, Dimension 3100, Digital Instruments, Santa Barbara, CA, USA) in tapping mode using silicon cantilever probes with a nominal tip radius of 10 nm under ambient laboratory conditions. The corresponding images of 10 × 10 µm^2^ size contain 512 data points in *x*-, and 256 lines in *y*-direction, with a nominal resolution in *z* of 20 pm (1,3 µm/16 bit in the full *z*-scale range). The data are displayed as two-dimensional color maps of the surface topography, using a common color scale for encoding topographic height (*z*-) variations.

AES: Auger electron spectroscopy was performed using a Scanning Auger Spectrometer PHI 700 (Physical Electronics GmbH, Feldkirchen/Ulvac-PHI, Inc., Japan). The measurements were carried out with a primary electron beam energy of 10 kV and a beam current of 9 nA. The sample was analyzed at a 30° tilt angle. Auger electron survey spectra were acquired with a step size of 1 eV, averaged over 10 cycles, each with a dwell time of 20 ms.

Raman spectroscopy: Horiba XploRA Plus equipped with a green 532 nm laser (100 mW) and an automated XYZ stage from Märzhäuser Wetzlar (Germany). The laser power was adjusted with a filter to 10 mW. An objective with 100x magnification was used for analysis. A map with an individual number of points was measured for each measurement while the distance between points was kept at 600 nm. The data was processed in LabSpec (Horiba) software. For the spectra the data was exported and plotted in Origin. For the unfunctionalized GO the intensities of G band (I_G_) and D band (I_D_) were taken at 1598 cm^-1^ and 1343 cm^-1^, respectively. The point defect distance (L_D_) was then estimated from the I_D_/I_G_ ratio.^[47]^

References

[47] L. G. Cancado, A. Jorio, E. H. M. Ferreira, F. Stavale, C. A. Achete, R. B. Capaz, M. V. O. Moutinho, A. Lombardo, T. S. Kulmala, A. C. Ferrari, Quantifying Defects in Graphene via Raman Spectroscopy at Different Excitation Energies *Nano Lett* **2011**, *11*, 3190–3196.

[54] P. Mrkwitschka, M. Sahre, E. Corrao, F. Pellegrino, V.-D. Hodoroaba, Wire-Print as a Sample Preparation Procedure Suitable for Accurate Morphological Characterization of Constituent Particles for Graphene-Related 2D-Materials *Microscopy and Microanalysis* **2025**, *31*.
